# Comparative Analysis of PRV-1 in Atlantic Salmon and PRV-3 in Coho Salmon: Host-Specific Immune Responses and Apoptosis in Red Blood Cells

**DOI:** 10.3390/microorganisms13051167

**Published:** 2025-05-21

**Authors:** Laura V. Solarte-Murillo, Sebastián Salgado, Tomás Gatica, Juan Guillermo Cárcamo, Thomais Tsoulia, Maria K. Dahle, Carlos Loncoman

**Affiliations:** 1 Laboratorio de Virología Molecular VIRIONLAB, Instituto de Bioquímica y Microbiología, Facultad de Ciencias, Universidad Austral de Chile, Valdivia 5090000, Región de Los Ríos, Chile; 2 Laboratorio de Bioquímica Farmacológica, Virología y Biotecnología, Instituto de Bioquímica y Microbiología, Facultad de Ciencias, Universidad Austral de Chile, Valdivia 5090000, Región de Los Ríos, Chile; 3 Interdisciplinary Center for Aquaculture Research, INCAR, Valdivia 5091000, Región de Los Ríos, Chile; 4 Departments of Aquatic Animal Health and Analysis and Diagnostics, Norwegian Veterinary Institute, 1433 Ås, Norwaymaria.dahle@vetinst.no (M.K.D.); 5 Department of Biotechnology, Fisheries and Economy, UiT Arctic University of Norway, 9019 Tromsø, Norway

**Keywords:** antiviral immune response, Atlantic salmon, coho salmon, red blood cells (RBCs), apoptosis, piscine orthoreovirus (PRV)

## Abstract

Fish red blood cells (RBCs) are nucleated, transcriptionally active, and key players in both gas transport and immune responses. They are the primary targets of *Orthoreovirus piscis* (PRV), the etiological agent of heart and skeletal muscle inflammation (HSMI), which includes three genotypes (PRV-1, PRV-2, and PRV-3), linked to circulatory disorders in farmed salmon. In Chile, PRV-3 affects the coho salmon (*Oncorhynchus kisutch*), but host–pathogen interactions remain poorly characterized. This study compared the interactions of PRV-3 in coho salmon and PRV-1 in Atlantic salmon (*Salmo salar*) using RBC infection models. RBCs were isolated from healthy juvenile salmon (n = 3) inoculated with either PRV-1 (Ct = 18.87) or PRV-3 (Ct = 21.86). Poly I:C (50 µg/mL) was used as a positive control for the antiviral response. Cells were monitored for up to 14 days post-infection (dpi). PRV-3 infection in coho salmon RBCs caused significant metabolic disruption, apoptosis from 7 dpi, and correlated with increasing viral loads. In contrast, PRV-1 infection in Atlantic salmon RBCs showed limited apoptosis and maintained cell viability. Coho salmon RBCs upregulated *rig-i*, *mx*, and *pkr* transcripts, indicating activation of the type I interferon pathway, whereas Atlantic salmon RBCs exhibited a more attenuated response. PRV-3 induced notable morphological changes in coho salmon RBCs, although neither PRV-3 nor PRV-1 caused hemolysis. These findings highlight species-specific differences in RBC responses to PRV infection and provide new insights into the pathogenesis of PRV-3 and PRV-1.

## 1. Introduction

Salmon farming has emerged as a vital source of nutritious food, bolstering the economies and communities in the regions where it is practiced. Norway stands out as the primary producer and exporter of Atlantic salmon (*Salmo salar*). Chile is also a prominent salmonid producer and exporter, particularly for the cultivation of coho salmon (*Oncorhynchus kisutch*) [1]. However, extensive mass-production and handling of salmonids in aquaculture has led to a heightened risk of infections [2]. Diseases originating from viruses are the second most significant cause of salmonid mortality and pose a serious threat to salmonid health and welfare, and production income. This threat is further intensified by the scarcity of effective vaccines and treatments, and the rapid evolution of viral genomes [3,4].

In recent years, several studies have investigated the genotypes of *Orthoreovirus piscis*—formerly and widely known as Piscine orthoreovirus (PRV)—and their role in triggering diseases [5,6,7]. PRV is a non-enveloped, double-stranded RNA virus that belongs to the *Spinareoviridae* family, *Orthoreovirus* genera, and *Orthoreovirus piscis* species [8]. PRV has an icosahedral, double-layered capsid approximately 70 nm in size. Its 23.3 kb genome consists of ten linear dsRNA segments, divided into four short (S1–S4), three medium (M1–M3), and three long (L1–L3) segments [9], encoding eleven proteins. Phylogenetic analyses of the S1 and M2 segments, which encode the outer capsid proteins σ3 and µ1, are commonly used for PRV classification [10]. The classification of PRV divides it into three distinct genotypes. PRV-1, first identified in 2010 [9], is the causative agent of heart and skeletal muscle inflammation (HSMI) in Atlantic salmon. PRV-2, on the other hand, was identified as the cause of erythrocytic inclusion body syndrome (EIBS) in coho salmon in Japan in 2016 [11]. PRV-3, which is responsible for HSMI-like diseases in rainbow trout [7,12] and coho salmon [13], has been correlated with jaundice syndrome in coho salmon in Chile [14,15]; however, the causality of PRV for jaundice syndrome has not been experimentally proven to date. PRV-3 is more closely related to PRV-1 (80% nucleotide identity) than to PRV-2 (73%), with PRV-1 and PRV-2 also sharing about 73% identity.

The PRV genotypes show differential host preference, and cause host-specific pathogenesis. In Atlantic salmon, PRV-1 is responsible for HSMI, a disease characterized by epicarditis, inflammation, and necrosis of the compact layer of the myocardium and red skeletal muscle. This condition is observed with no anemia, but with a transient dip in hemoglobin. PRV-1 infection in Atlantic salmon leads to a persistent phase, with the viral genome detectable for at least 65 weeks in the heart, muscle, blood, spleen, and kidney, despite low replication and protein production [5]. The mortality rate due to PRV-1 infection in Atlantic salmon aquaculture is 0–20% in Norway [16] and 0–5% in Chile [17]. However, the prevalence of PRV-1 in farmed Atlantic salmon exceeds 80%. The disease typically emerges some weeks after the transfer to the sea. Additionally, salmon are often infected already in freshwater farms [18].

In Japan, coho salmon infected with PRV-2 have routinely displayed the presence of erythrocytic inclusion bodies and anemia [11]. These salmonids exhibit necrotic muscle fiber degeneration in the cardiac ventricles and atria, as well as jaundice caused by hyperbilirubinemia and bilirubin accumulation in the liver [11]. The PRV-2 genotype has primarily been identified in Japan, causing a total mortality rate in coho salmon below 20% [19]. More recently, a diagnostic investigation of disease in a cohort of coho salmon in Alaska also tested positive for PRV-2 [20].

In rainbow trout infected with PRV-3, the primary affected organ is the heart, which exhibits endocarditis, myocarditis, and epicarditis, as well as necrosis of cardiomyocytes and lesions in skeletal muscle [12]. Additionally, other organs, such as the liver, appear pale and yellowish, while the kidney and spleen show inflammation with hemosiderosis [12]. Clinical signs in coho salmon infected with PRV-3 include a yellow liver, pale heart, biliary cholestasis, ascites, clotted blood in the abdominal cavity, and, in some cases, spinal fracture and kidney rupture [13,21]. Histologically, hemosiderosis is absent, but erythrophagocytosis occurs in the spleen [13]. PRV-3 infection leads to severe anemia and does not cause persistent infection in the host [7,12].

The results of experimental studies suggest that PRV-3 can infect Atlantic salmon without causing disease and have the potential to provide cross-protection against HSMI caused by PRV-1 [6]. The absence of a suitable cell culture system for propagating PRV [22] presents a significant challenge for studying virus–host interactions in vitro and for eventually producing inactivated viral vaccines. PRV exhibits a preference for infecting erythrocytes or red blood cells (RBC). Within these cells, the virus reaches its peak replication before spreading to secondary organs [23,24]. Although the main role of RBCs is gas exchange, these cells have recently gained attention for their participation in the immune response to infections caused by bacteria [25,26], fungi [27], and viruses [24,28]. This involves pathways linked to the innate immune response, apoptosis, reactive oxygen species (ROS) production, and the role of these cells in presenting antigens, which can mediate the activation of the adaptive immune response.

The infection of Atlantic salmon RBC by PRV-1 triggers the activation of the type I interferon signaling pathway and antiviral effectors such as *pkr*, *mx*, *isg15*, and *viperin* through pathogen-associated molecular patterns (PAMPs) like the dsRNA genome [24,29]. Although similar outcomes are believed to occur in other host species’ RBC infected with PRV, hypotheses suggest that distinct hematological effects of PRV infection in different species (such as anemia, hemosiderosis, and erythrophagocytosis) indicate that erythrocytic responses and tolerance to PRV infection differ, potentially leading to various pathological outcomes [30]. In this study, we focused on investigating and comparing the RBC immune response and apoptosis triggered by the infection of PRV-3 in coho salmon RBC vs PRV infection in Atlantic salmon RBC. Understanding how these viruses interact with RBC from their preferred host species may be key to unlocking new features of their pathogenesis.

## 2. Material and Methods

### 2.1. Overall Description

Two ex vivo infection experiments were conducted using red blood cells (RBC). In the first experiment (I), coho salmon RBC (csRBC) were infected with the PRV-3a genotype, which was isolated from Chilean coho salmon (isolate PRV-59+) [15]. In the second experiment (II), Atlantic salmon RBC (asRBC) were infected with PRV-1b, which was isolated from Norwegian Atlantic salmon (isolate NOR2012-V3621). Experiment I was carried out at the Laboratorio de Virología Molecular at the Facultad de Ciencias, Universidad Austral de Chile, located in Valdivia, Chile. Experiment II was conducted at the Norwegian Veterinary Institute in Ås, Norway. The experimental protocols for both experiments are provided below.

### 2.2. Isolation and Purification of the PRV Inoculums

The PRV-3a inoculum used in experiment I was obtained from an outbreak of jaundice syndrome affecting coho salmon during the fattening stage in the sea in southern Chile [15]. Fish from the outbreak in Chile exhibited clinical signs of jaundice and tested negative for other prevalent viral and bacterial diseases. Inoculum clarification was performed following the protocol described by Pham et al. [22] with some modifications. In summary, a positive pool of spleen and heart for PRV-3 was diluted 1:5 in Leibovitz’s L-15 medium (Life Technologies) with 1X antibiotic-antimycotic (Gibco, Valdivia, Chile) to homogenize using a Polytron, ThermoFisher, Valdivia, Chile. Homogenized samples were sonicated on ice with eight 30 s pulses at 25 Hz, with 10 s of rest between them. The lysed tissue was centrifuged twice at 5000 g for 10 min at 4 °C to remove cellular debris. The supernatant was filtered using 0.2 µm syringe filters between each centrifugation. Finally, the clarified supernatant was purified using a cesium chloride (CsCl) gradient. The CsCl gradient purification protocol was performed as described by Wessel et al. with minor modifications. Briefly, two CsCl solutions were prepared: a heavy solution (1.45 g/mL) and a light solution (1.2 g/mL) in Tris HCl (pH 7.8). The solutions were layered in a 2 mL ultracentrifuge tube, with the heavy solution at the bottom, the light solution in the middle, and 2 mL of the clarified inoculum on top. The mixture was then centrifuged at 100,000× *g* for 12 h in a Himac CP90WX Hitachi centrifuge with a P90AT rotor. After centrifugation, two well-defined bands were observed: the upper band, primarily composed of defective infectious particles, and the lower band, containing the infectious viral particles. The lower band was carefully collected with a 21G syringe needle from the top of the centrifuge tube, transferred to a new tube, and subjected to further centrifugation at 100,000× *g*. The viral pellet was resuspended in L-15 culture medium at pH 7.6 and stored at −80 °C until use.

The PRV-1 inoculum for experiment II was obtained from blood samples collected in a previous cohabitation trial involving Atlantic salmon, four weeks post-infection. The inoculum used was an isolate from a Norwegian field outbreak of HSMI in 2012 (NOR2012-V3621). The complete genome sequence of this isolate (NOR2012-V3621) was reported in 2017. The inoculum was prepared and purified as described previously. Briefly, the blood pellet was diluted 1:10 in HO buffer and sonicated three times on ice for 20 s at 50% amplitude (60 s rest in between). The blood samples were then mixed with 10% (*w*/*v*) sodium desoxycholate (DOC) and Vertrel XF for homogenization and sonication. The aqueous and organic phases were separated by centrifugation at 9000× *g*/10 min/4 °C to collect the aqueous phase. The top aqueous phase was collected and layered onto a 1.22–1.45 g/mL CsCl gradient and centrifuged for >12 h at 30,000 rpm using the Sorvall WX+ Ultracentrifuge, TH 641 Rotor, Thermo Fisher. After centrifugation, the tubes were punctured using a G21 needle, and the fractions were collected by gravitational drop to measure their density using a refractometer and converted to g/cm^3^. The PRV floating density in CsCl was estimated to be approximately 1.36 g/mL. Finally, the purified samples were injected into Slide-A-Lyzer Cassettes (G2 10 kDa MWCO, Thermo Fisher) and dialyzed three times against D-buffer. Purified virus samples were collected from dialysis cassettes, and glycerol was added to a final concentration of 15% and stored at −80 °C until use.

### 2.3. Blood Sampling and Isolation of RBC

For experiment I, three healthy juvenile coho salmon (weight: 80–150 g) were sourced from a fish farm in Cutipay, Valdivia, Chile. The fish were euthanized with an overdose of benzocaine and blood was promptly drawn from the caudal vein using heparinized syringes (1 mL). Individual blood samples were transported in BD Vacutainer tubes containing Lithium Heparin (75 USP Units) to the Molecular Virology Laboratory of the Institute of Biochemistry and Microbiology, Faculty of Sciences at the Universidad Austral de Chile, maintaining a cold chain at 4 °C, and processed on the same day. In parallel, heart, liver, spleen, and anterior kidney samples were collected, labeled appropriately, and transported to the laboratory in 1.5 Eppendorf tubes with RNA later (Thermo Fisher). These samples were placed in a container with liquid nitrogen and subsequently stored at −80 °C until analysis.

For both experiments, RBC were isolated and purified using the protocol proposed by Finstad et al. with Percoll 51% (Sigma-Aldrich, Valdivia, Chile). Briefly, the blood was diluted 1:20 in 1X PBS. The diluted blood was layered on the Percoll gradient and centrifuged at 500× *g*/20–30 min/4 °C at a low break. RBC were collected from the bottom of the gradient and the supernatant was discarded. Isolated RBC were washed twice with PBS and resuspended in L-15 medium supplemented with antibiotics and 2% serum.

### 2.4. RBC Culture and Treatments

For experiment I, cells were seeded in 24-well Falcon Primaria™ culture plates (10 × 10^6^ cells/mL) in L-15 medium (Life Technologies, Valdivia, Chile) supplemented with 2% fetal bovine serum and 1X antibiotic–antimycotic solution (Gibco™). The cells were cultured for 24 h at 15 °C with constant shaking (225 rpm) prior to stimulation. The RBC were then exposed to two treatments and a control group: PRV-3 (100 μL/mL) and Poly I:C (50 µg/mL; positive control dsRNA). Biological triplicates were included for each treatment and control group.

For experiment II, the cells were seeded in 12-well Falcon Primaria™ culture plates (20 × 10^6^ cells/mL) in L-15 medium (Life Technologies) supplemented with fetal bovine serum (2%) and gentamycin (50 µg/mL). The cells were cultured for 24 h at 15 °C with constant shaking (225 rpm) before stimulation. The RBC were exposed to two treatments and a control group: PRV-1 (5 μL/mL) and Poly I:C (50 µg/mL; positive control dsRNA). Biological triplicates were included for each treatment and control group.

After 24 h of incubation, the plates were centrifuged at 500× *g*/5 min/4 °C to remove the supernatant and washed in L-15 medium to completely remove the excess non-adsorbed virus. Subsequently, RBC were incubated for 14 days at 15 °C with constant shaking. Samples were harvested at 1-, 3-, 7-, and 14-days post infection (dpi) to measure cell viability using the Trypan blue exclusion method with Trypan blue Solution, 0.4% (Gibco™), and the Alamar blue assay (Invitrogen™, Valdivia Chile) following the manufacturer’s instructions. Additionally, 20 × 10^6^ cells samples were harvested and centrifuged at 800× *g* for 5 min to separate the RBC pellet from the supernatant and perform RNA extraction for viral quantification and gene expression analysis of RBC with the respective treatments. RBC cultures for both experiments were examined by phase contrast microscopy for appearance and morphological changes.

### 2.5. Alamar Blue Assay

Initial studies were conducted to determine the optimal incubation time and number of cells required. A calibration curve was established for this purpose using various concentrations of DMSO (0.1%, 1%, 5%, 10%, 20%, and 30%) (Figure S1). For the cell viability analysis with PRV treatments, cells were seeded in a 96-well plate at a density of 1×10^5^ cells/well with only L-15 medium and a 10% (*v*/*v*) solution of Alamar blue reagent (Thermo Fisher, Valdivia, Chile). RBC were then incubated for 72 h at 15 °C and fluorescence was measured (excitation at 540 nm and emission at 590 nm) at 1, 7, and 14 dpi. The measurement for experiment I was conducted using the Biotek Synergy 2 Multi-Mode Plate Reader (Agilent Technologies, Valdivia, Chile), and for experiment II, the SpectraMax^®^ i3x Multi-Mode Microplate Reader (Agilent Technologies, Valdivia, Chile), was employed. Cell viability was normalized to that of the control group, with regular media without any treatment serving as blank subtraction.

### 2.6. Total RNA Extraction, cDNA Synthesis, and Relative Gene Expression in RBC

For experiment I, total RNA was extracted from pelleted csRBC (200 µL) using a Total RNA Mini Kit (Tissue) (Qiagen, Valdivia, Chile) according to the manufacturer’s instructions and digested with DNase to eliminate genomic DNA contamination. RNA concentration and quality were evaluated using a MaestroNano Micro-Volume Spectrophotometer (Agilent Technologies, Valdivia, Chile). Complementary DNA (cDNA) was synthesized from 200 ng of total RNA in a 20 μL reaction volume using Oligo dT20 (Thermo Fisher, Valdivia, Chile) and the reverse transcriptase RevertAID (Thermo Fisher) according to the manufacturer’s instructions. Two negative controls were used: a no-template control (NTC) and no-enzyme control (RTN). Finally, the cDNA was diluted 1:4 and stored at −20 °C until use. Quantitative PCR based on SYBR green was performed to evaluate the transcription profiles of the target antiviral genes using a LineGene 9640 real-time PCR system (BIOER, Valdivia, Chile). The reaction included: 4 μL of 5 × HOT FIREPol EvaGreen qPCR Mix Plus (ROX) (Solis BioDyne, Valdivia, Chile), 0.5 μL of each forward and reverse primer (both 10 μmol/L), 2 μL of diluted cDNA template (5 ng of input), and sterile distilled water free of nucleases up to a final volume of 20 μL. Thermocycling for all reactions was 12 min at 95 °C, followed by 40 cycles of 15 s at 95 °C, 20 s at 60 °C, and 20 s at 72 °C. The fluorescence and melting curves were monitored at the end of each cycle to confirm the amplification of a single product for each reaction. Relative mRNA expression was normalized against that of the *β-actinA* gene using the ΔΔCt method, where ΔCt was determined by subtracting the *β-actinA* value from the target Ct. The *β-actinA* gene was selected as the reference gene according to the MIQE guidelines [31], given that no statistical differences were detected among Ct values obtained for *β-actinA* in the different samples (Figure S2A). The primers used are listed in Table 1.

For experiment II, total RNA was extracted from pelleted asRBC (200 µL) using an automated MagNA Pure 96 Cellular RNA Large Volume Kit (Roche), according to the manufacturer’s protocol. RNA concentration and quality were evaluated using a Multiskan Sky Microplate Spectrophotometer (Thermo Fisher, Ås, Norway). cDNA was synthesized from 200 ng of total RNA in a 20 μL reaction volume using the QuantiTect Reverse Transcription Kit (Qiagen, Ås, Norway) containing gDNA wipeout buffer according to the manufacturer’s instructions. Two negative controls were used, a no-template control (NTC) and a no-enzyme control (RTN). Finally, the cDNA was diluted 1:4 and stored at −20 °C until use. Quantitative PCR based on SYBR green was performed to evaluate the transcription profiles of the target antiviral genes using the BioRAD CFX 384 instrument. The reaction mixture included 5 μL of SsoAdvanced Universal SYBR Green Supermix (Cat No. 172–5271 (Bio-Rad, Ås, Norway), 0.5 μL of each forward and reverse primer (both 10 μmol/L), 2 μL of diluted cDNA template (5 ng of input), and sterile distilled water up to a final volume of 10 μL. The cycling parameters for all reactions were 30 s at 95 °C, followed by 40 cycles of 15 s at 95 °C, and 30 s at 60 °C. Fluorescence and melting curves were monitored at the end of each cycle to confirm amplification of a single product for each reaction. Relative mRNA expression was normalized to the *ef1α* gene using the ΔΔCt method, where ΔCt is determined by subtracting the *ef1α* value from the target Ct. *ef1α* was selected as reference gene according to the MIQE guidelines [31], given that no statistical differences were detected among Ct values obtained for *ef1α* in the different samples (Figure S2B). Primers are listed in Table 1.

### 2.7. PRV Detection and Kinetics in Salmonid RBC

For experiment I, viral RNA was purified from RBC total RNA using the Viral Nucleic Acid Extraction Kit II with DNAse (Geneaid, Valdivia, Chile) following the standard protocol. RNA was eluted in 30 μL of free-nuclease water and quantified in the Maestro Nano Micro-Volume Spectrophotometer. RT-qPCR for PRV-3 kinetics targeting the S1 segment (Table 1) was performed using the Brilliant II SYBR Green qRT-PCR 1-Step Master Mix (Agilent Technologies, Valdivia, Chile) with 100 ng (up to 5 μL) of RNA per reaction in duplicate. The reaction conditions included: 6 μL of 2× SYBR Green QRT-PCR master mix, 0.5 μL of each forward and reverse primer (both 10 μmol/L), 0.5 μL of RT/RNase block enzyme mixture, and free-nuclease water until 12.5 μL of total reaction volume. The template was denatured at 95 °C for 5 min using a LineGene 9640 real-time PCR system (Bioer, Valdivia, Chile). The cycling parameters were set to 30 min at 50 °C, 10 min at 95 °C, and 40 cycles for 30 s at 95 °C, and 1 min at 60 °C.

For experiment II, RT-qPCR for PRV-1 kinetics targeting the S1 segment (Table 1) was performed using the Qiagen OneStep RT-PCR kit (Qiagen, Ås, Norway) with 5 μL of 20 ng/μL total RNA (100 ng of input) in each reaction tube, following the reaction conditions recommended by the manufacturer. The reaction conditions included: 4 μL of Qiagen OneStep RT-PCR buffer, 0.8 μL of dNTP (10 mM), 0.5 μL of each forward and reverse primer (both 10 μmol/L), 0.4 μL of the probe (10 μmol/L), 1 μL of MgCl2 (25 mM), and free-nuclease water until a final volume of 20 µL. The RNA template was then denatured at 95 °C for 5 min. The reverse transcription (RT) step was conducted at 50 °C for 30 min, followed by 95 °C for 15 min, 40 cycles of 94 °C for 30 s, and 60 °C for 1 min in the CFX96 Touch Real-Time PCR Detection System (Bio-Rad, Ås, Norway).

The specificity of the assay was confirmed by melting curve analysis, and negative controls (NTC) were included to confirm the absence of contamination. The Ct values of PRV-1 and PRV-3 were normalized to those of the respective housekeeping genes (Table 1).

### 2.8. Flow Cytometry

To determine whether viral infection induced apoptosis in RBC, phosphatidylserine (PS) externalization on the RBC surface following PRV infection was assessed using flow cytometry with the Annexin V-Alexa Fluor 488 staining kit. Flow cytometric analyses were performed using the BD FACSCanto™ II (BD Biosciences, Valdivia, Chile) flow cytometer for experiment I, and the Acea Biosciences NovoCyte 2000 Flow Cytometer for experiment II. RBC size and density were assessed using forward and side-angle scattering (FSC vs. SSC). Compensation was performed using four controls: unstained control cells (Q4 quadrant), apoptotic cells stained with Annexin V (Q3 quadrant), and dead cells stained with propidium iodide (PI) (Q1 quadrant) (Figure S3A). RBC were subjected to an apoptosis control procedure based on the method proposed by Li et al. [33], which involved incubation in an L-15 medium containing 2% serum and antibiotics with FeSO4/H2O2 (40 μM/20 μM) for 18 h (Figure S3B). Cell death was induced by exposing the RBC to 5% DMSO for 8 h (Figure S3C). For this analysis, the Dead Cell Apoptosis Kit with Annexin V Alexa Fluor™ 488 and Propidium Iodide (Invitrogen) was used, according to the manufacturer’s instructions. Briefly, RBC were washed with cold PBS buffer (pH 7.4) and resuspended at a density of 1 × 10^6^ cells/well in 100 μL of 1X annexin-binding buffer. Next, 5 μL of Alexa Fluor™ 488 Annexin V and 1 μL of 100 µg/mL PI were added to the cell suspension. The cells were incubated at room temperature for 15 min. After the incubation period, 400 μL of 1X annexin-binding buffer was added, and the cells were kept on ice for flow cytometry analysis. Flow cytometry data were processed and analyzed using FlowJo^TM^ v10.8 Software (BD Life Sciences, Ashland, OR, USA) [34].

### 2.9. Immunofluorescence Microscopy

Confirmation of Annexin V and PI staining was performed under fluorescence microscopy according to the kit protocol instructions (Invitrogen) (Figure S4). For experiment I, the cells were mounted to glass slides and cover slips and images were captured using an upright light microscope (Olympus BX43), fluorescent light source (Olympus UHGLGPS) at 20X magnification. For experiment II, the stained cells were mounted to a 96-well plate and images were captured in the Zeiss AXIO observer A1, invert fluorescence microscope at 40X magnification.

### 2.10. Statistical Analysis

Data from three biological replicates of both experiments were analyzed using GraphPad Prism V10 software. Two-way ANOVA (with Tukey’s post hoc test for multiple comparisons) was used for statistical analysis. The two independent variables considered for each experiment were: (i) the treatment groups (control, Poly I:C, PRV infection) and (ii) the time points during the ex vivo culture. The dependent variables included the fold change for the qPCR assay, the percentage of cell viability for the Alamar blue and Trypan blue assays, and the percentage of annexin binding for the apoptosis assay. The *p* values less than 0.05 were considered significant (* *p* ≤ 0.05, ** *p* ≤ 0.01, *** *p* ≤ 0.001, and **** *p* ≤ 0.0001).

## 3. Results

### 3.1. PRV-3 and PRV-1 Genotypes Infect Salmonids RBC Ex Vivo

**Figure 1:**
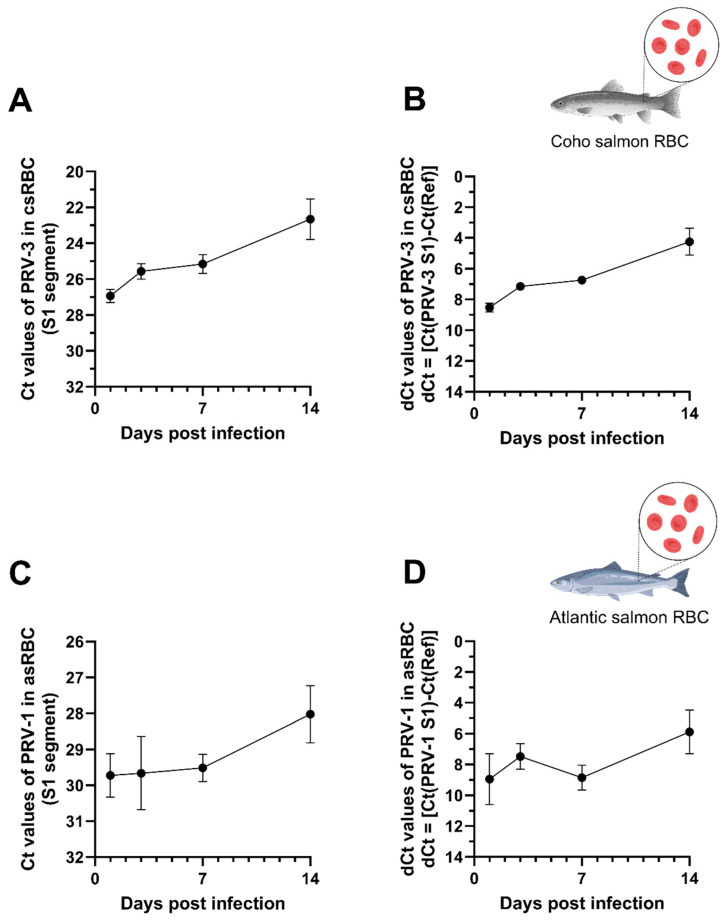
PRV kinetics in salmonid RBC ex vivo. (**A**) PRV-3 Ct values in coho salmon RBC (csRBC) and (**B**) normalized to the housekeeping gene (*β-ActinA*). (**C**) PRV-1 Ct values in Atlantic salmon RBC (asRBC) and (**D**) normalized to the housekeeping gene (*ef1α*). Bars represent the mean ± standard error (SE) (n = 3). PRV Ct values for the S1 segment were measured by RT-qPCR in RBC at 1, 3, 7, and 14 days post-infection (dpi).

To assess the infectivity of PRV-3 in csRBC ex vivo, the cells were exposed to the isolate PRV-59+ (viral genome:cells 1:2) with an inoculate Ct value of 21.86 ± 0.29. To assess the infectivity of PRV-1 in asRBC ex vivo, the cells were exposed to the isolate NOR2012-V3621 (viral genome:cells 1:1) with an inoculate Ct value of 18.87. For experiment I, csRBC showed a Ct value of 26.94 ± 0.51 at 1 dpi and exhibited a decreasing Ct value over time, reaching the lowest Ct value at 14 dpi (22.66 ± 1.59 (Figure 1A,B).

A similar tendency was observed for experiment II; asRBC showed a Ct value of 29.72 ± 0.85 (assay targeting PRV-1 S1 segment) at 1 dpi, and the lowest Ct value at 14 dpi (28.02 ± 1.1) (Figure 1C,D).

For both trials, the cell entry efficiency of the PRV infection was not optimal, as indicated by the higher Ct value at 1 dpi in the RBC fraction compared to the initial inoculum Ct value (Figure 1). However, the PRV-1 dCt values normalized with the housekeeping genes demonstrated an increase in PRV infection in salmonid RBC over time. Specifically, for csRBC the PRV-3 increase was linear until 14 dpi (Figure 1B), whereas asRBC displayed a gradual PRV-1 increase from 1 dpi to 3 dpi and from 7 dpi to 14 dpi (Figure 1D).

### 3.2. PRV-3 Affects Cell Metabolism but Does Not Induce Cell Lysis in the Coho Salmon RBC Ex Vivo

**Figure 2:**
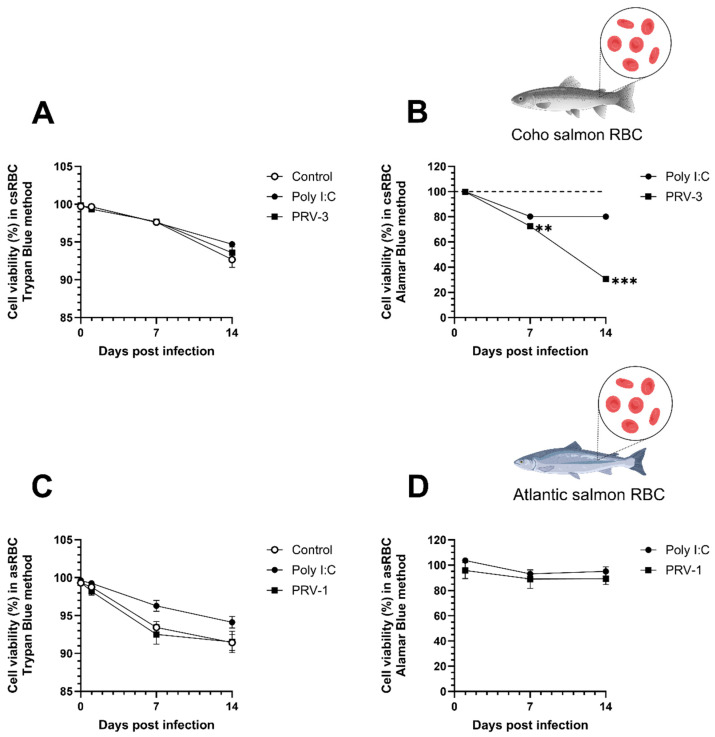
RBC viability assays after PRV infection. (**A**) Trypan blue viability assay and (**B**) Alamar blue viability assay performed on coho salmon RBC (csRBC) after treatment with PRV-3 and Poly I:C (50 µg/mL) for 14 days. (**C**) Trypan blue viability assay and (**D**) Alamar blue viability assay performed on Atlantic salmon RBC (asRBC) after treatment with PRV-1 and Poly I:C (50 µg/mL). Bars represent the mean ± standard error (SE) (n = 3). Significant differences are represented by ** *p* < 0.01, and *** *p* < 0.001, two-way ANOVA, Tukey’s post hoc analysis.

The Trypan blue viability assay showed no significant differences in RBC viability between control cells and cells infected with PRV-3, PRV-1, or those treated with Poly I:C (Figure 2A,C). All groups analyzed exhibited a slight decline in cell viability from 1 dpi to 14 dpi, reaching approximately 91% to 93% by the end of the ex vivo culture.

We found using the Alamar blue (Thermo Fisher, Valdivia, Chile). viability assay that csRBC metabolic activity decreased significantly from 7 dpi to 14 dpi when infected with PRV-3 (Figure 2B), but this was not found in asRBC infected with PRV-1 (Figure 2D).

### 3.3. PRV-3 Infection Induces Apoptosis in Coho Salmon RBC Ex Vivo

**Figure 3:**
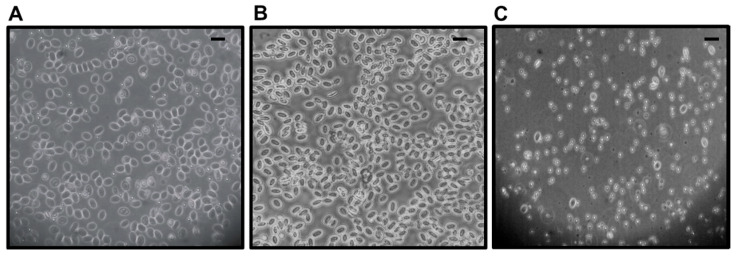
Morphological changes in coho salmon RBC (csRBC) ex vivo at 14 dpi. Images were taken using a phase contrast microscope at 20X magnification (Olympus, Valdivia, Chile). Solid black line represents scale bar to represent 15 um (**A**) Control csRBCs show an elliptical shape with intact cytoplasm and preserved morphology. (**B**) Poly I:C-treated csRBCs (50 µg/mL) display morphology similar to controls, with no significant structural changes. (**C**) PRV-3-infected csRBCs exhibit senescence-like features, including a rounded shape, reduced cell size, and cytoplasmic depletion, indicating a response to viral infection.

As previously described, RBC did not display apparent membrane rupture following PRV infection. However, a noticeable effect on cell metabolism was observed in csRBC infected with PRV-3 (Figure 2B). The observed morphological changes entailed a transition from elliptical to rounded shape, a reduction in size, and a depletion of the cytoplasm (Figure 3). In contrast, asRBC did not exhibit morphological changes during PRV-1 infection (Figure S5).

**Figure 4:**
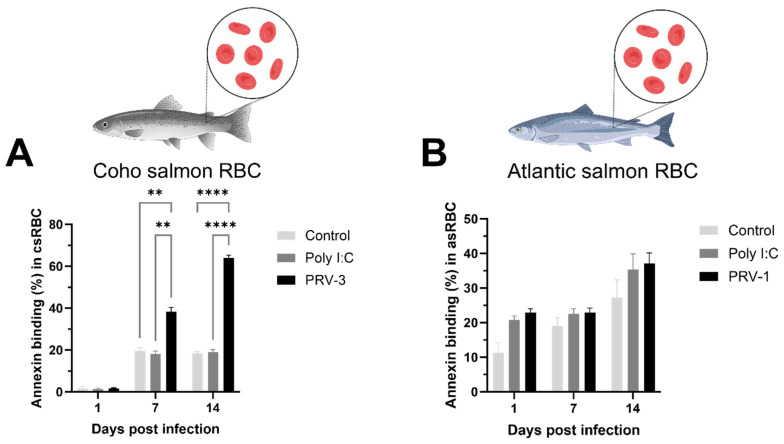
Annexin V binding in RBC ex vivo. (**A**) Percentage of Annexin V-positive cells in csRBC infected with PRV-3 and Poly I: C. (**B**) Percentage of Annexin V-positive cells in asRBC infected with PRV-1 and Poly I:C. Bars represent mean ± standard error for each treatment (n = 3) at 1, 7, and 14 dpi. Asterisks represent significant differences, represented as ** *p* < 0.01 and **** *p* < 0.0001, two-way ANOVA, Tukey’s post hoc analysis.

The percentage of csRBC detected with Annexin V binding to PS on the surface was significantly increased at 7 dpi and 14 dpi, suggesting an increase in early apoptotic cells after PRV-3 infection (Figure 4A). In contrast, the percentage of asRBC with Annexin V binding showed no significant differences over time or between the treatments (Figure 4B).

The csRBC infected with PRV-3 showed 64.1% ± 1.8 early apoptotic cells at 14 dpi (Figure S6C), while the control (Figure S6A) and Poly I:C-treated (Figure S6B) groups displayed 18.3% ± 1.3 and 19.1% ± 1.6 early apoptotic cells, respectively (quadrant Q3). In the asRBC, the percentage of early apoptotic cells did not significantly differ between the control (Figure S6D), Poly I:C (Figure S6E), and PRV-1 treatment groups (Figure S6F). Interestingly, in both experiments, the apoptotic state induced by Poly I:C or PRV infection was sustained over time and did not overlap with cell membrane destruction or necrosis (quadrant Q1) (Figure S6).

### 3.4. Purified PRV-3 Elicits Strong Antiviral Immune Response in Coho Salmon RBC Ex Vivo

The expression levels of the housekeeping genes *β-actinA* and *ef1α* for csRBC and asRBC remained stable across all time points following PRV infection and treatment with Poly I:C (50 µg/mL), with no statistically significant differences observed between treatments in each experiment (Figure S2).

**Figure 5:**
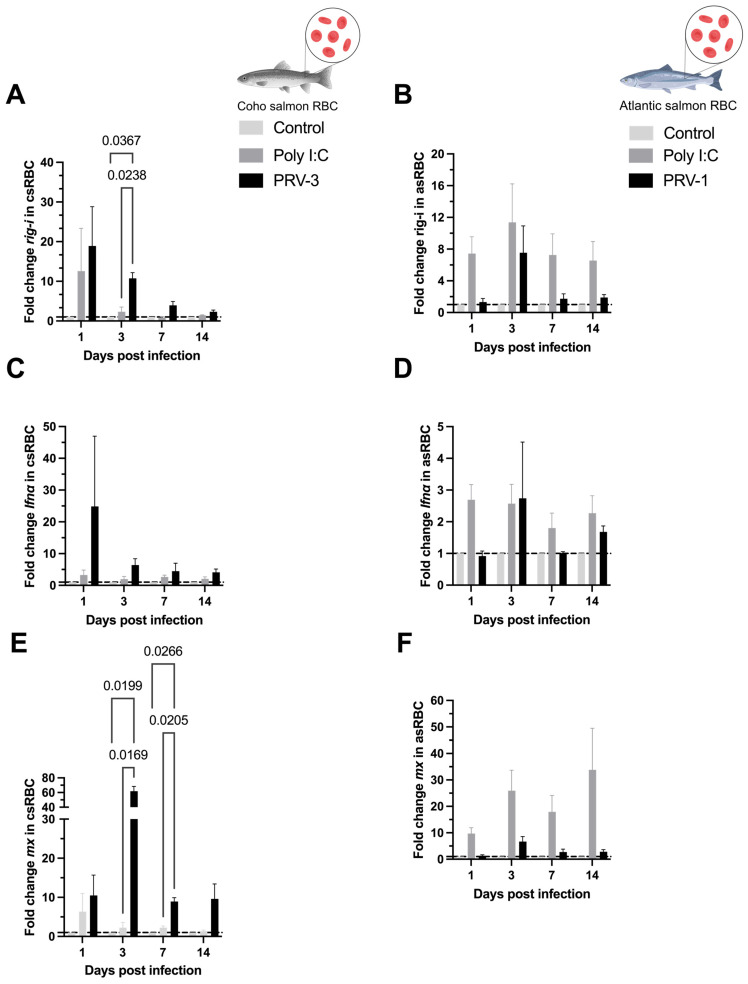
Expression of antiviral immune response transcripts (mRNA) in coho salmon RBC (csRBC) (**A**,**C**,**E**) and Atlantic salmon RBC (asRBC) (**B**,**D**,**F**) ex vivo. The expression levels of *rig-i* (**A**,**B**), *ifnα* (**C**,**D**), and *mx* (**E**,**F**) were assessed using RT-qPCR. Data are presented as the mean ± standard error for each treatment: Control, Poly I:C (50 µg/mL), PRV-1 infection in asRBC (n = 3), and PRV-3 infection in csRBC (n = 3) at 1, 3, 7, and 14 dpi. The fold change is represented based on the control (line) using the ΔΔCT method and normalized against *β-actinA* for csRBC and *ef1α* for asRBC. Significant differences are represented by two-way ANOVA, Tukey’s post hoc analysis.

In csRBC infected with PRV-3 the expression of the *rig-i* transcript was upregulated at 3 dpi compared to the control and Poly I:C groups (10.76 ± 1.15) (Figure 5A). In asRBC infected with PRV-1, the *rig-i* transcript showed no significant differences between the groups over time (Figure 5B). The gene expression of alpha interferon (*ifnα*) was not upregulated in neither experiment I nor II (Figure 5C,D). The csRBC displayed upregulation of *mx* antiviral effector transcripts at 3 and 7 dpi (61.97 ± 5.25 and 8.95 ± 0.79) (Figure 5E).

**Figure 6:**
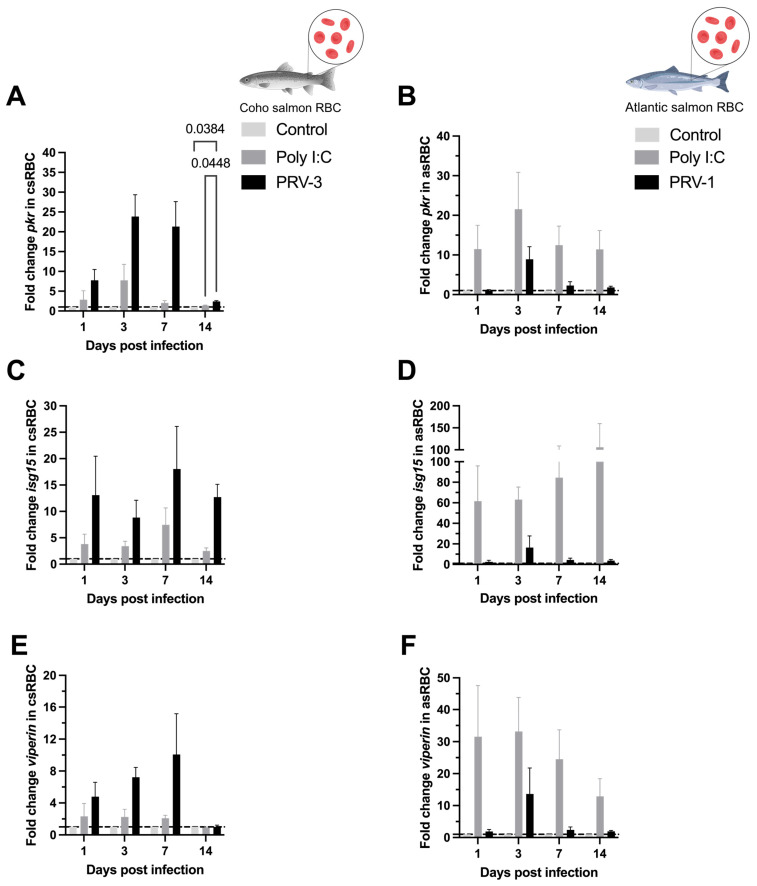
Expression of antiviral immune response transcripts (mRNA) in coho salmon RBC (csRBC) (**A**,**C**,**E**) and Atlantic salmon RBC (asRBC) (**B**,**D**,**F**) ex vivo. The expression levels of *pkr* (**A**,**B**), *isg15* (**C**,**D**), and *viperin* (**E**,**F**) were assessed using RT-qPCR. Data are presented as the mean ± standard error for each treatment: Control, Poly I:C (50 µg/mL), PRV-1 infection in asRBC (n = 3), and PRV-3 infection in csRBC (n = 3) at 1, 3, 7, 14 dpi. The fold change is represented based on the control (line) using the ΔΔCT method and normalized against *β-actinA* for csRBC and *ef1α* for asRBC. Significant differences are represented by two-way ANOVA, Tukey’s post hoc analysis.

The relative expression of *pkr* was only significantly upregulated at 14 dpi in csRBC infected with PRV-3 (2.41 ± 0.17), although a non-significant increase in *pkr* transcript was observed after 1 dpi in csRBC (Figure 6A). Neither up- nor down-regulation of *pkr* was observed in asRBC (Figure 6B). The antiviral effectors *isg15* and *viperin* did not display significant up- or down-regulation (Figure 6C–F).

**Figure 7:**
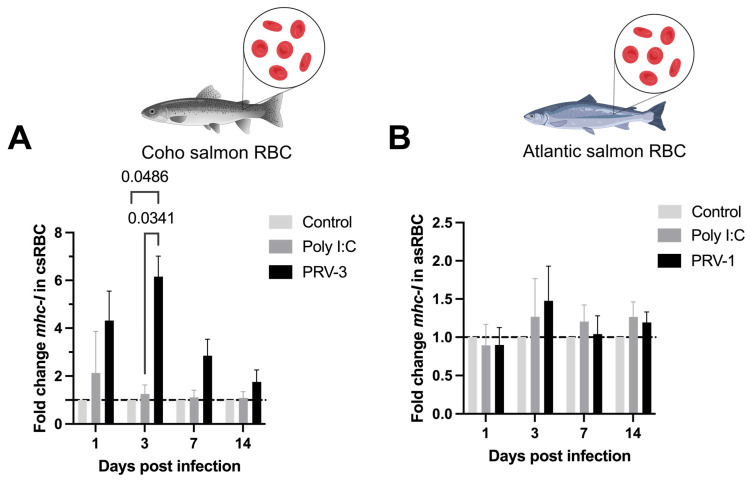
Expression of antigen presentation transcripts for *mhc-i* (mRNA) in coho salmon RBC (csRBC) (**A**) and Atlantic salmon RBC (asRBC) (**B**) ex vivo. The expression levels of *mhc-i* were assessed using RT-qPCR. The data are presented as the mean ± standard error for each treatment: Poly I:C (50 µg/mL), and the PRV-1 infection in asRBC (n = 3), and the PRV-3 infection in csRBC (n = 3) at 1, 3, 7, and 14 dpi. The fold change is represented based on the control (line) using the ΔΔCT method and normalized against *β-actinA* for csRBC and *ef1α* for asRBC. Significant differences are represented by two-way ANOVA, Tukey’s post hoc analysis.

PRV-3-infected csRBC significantly upregulated *mhc-i* transcripts (6.15 ± 0.70) at 3 dpi as shown in Figure 7A.

### 3.5. Gene Expression of Caspases and Apoptotic Regulators During PRV-3-Induced Apoptosis in Coho Salmon RBC

**Figure 8:**
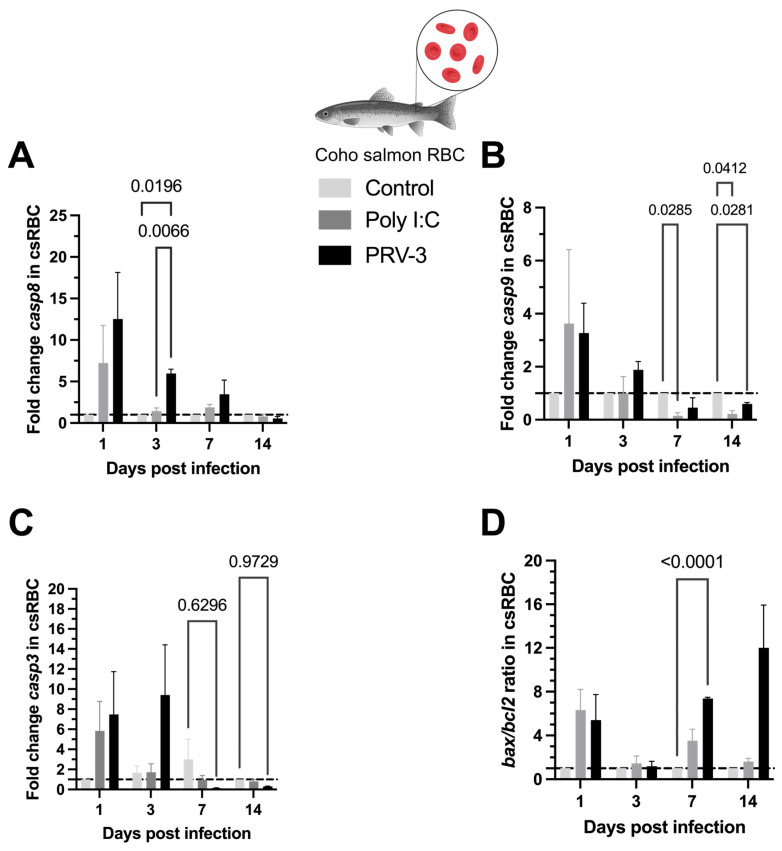
Expression of caspases transcripts and ratio *bax*/*bcl2* (mRNA) in coho salmon RBC (csRBC) ex vivo. The expression levels of *casp8* (**A**), *casp9* (**B**), *casp3* (**C**), and the ratio *bax*/*bcl2* (**D**) were assessed using RT-qPCR. Data are presented as the mean ± standard error for each treatment: Control, Poly I:C (50 µg/mL), PRV-3 infection in csRBC (n = 3) at 1, 3, 7, and 14 dpi. The fold change is represented based on the control using the ΔΔCT method and normalized against *β-actinA*. Significant differences are represented by two-way ANOVA, Tukey’s post hoc analysis.

The expression of *casp8* transcript in csRBC was upregulated by PRV-3 at 3 dpi compared to the control and Poly I:C groups (5.97 ± 0.42) (Figure 8A). In contrast, the expression of *casp9* transcripts was downregulated at 7 dpi and 14 dpi by Poly I:C stimulation compared to the control group (0.15 ± 0.09 and 0.22 ± 0.10), and at 14 dpi by PRV-3 infection (0.60 ± 0.04) (Figure 8B). The *casp3* transcripts were also downregulated in infected scRBC at 7 dpi and 14 dpi (0.14 ± 0.03 and 0.31 ± 0.02) (Figure 8C). Finally, the ratio of *bax*/*bcl2* was significantly upregulated at 7 dpi in csRBC infected with PRV-3 (7.38 ± 0.09) (Figure 8D).

## 4. Discussion

This study highlights the distinct host–pathogen interactions in the homotypic infection of PRV-1 in Atlantic salmon RBC and PRV-3 in coho salmon RBC under ex vivo culture conditions. While PRV-1 did not induce apoptosis in asRBC, PRV-3 significantly altered cellular metabolism and promoted early apoptosis in csRBC. Notably, this is the first report of purified PRV-3 infection in coho salmon RBC ex vivo, demonstrating the activation of the host’s antiviral immune response. The differential outcomes between PRV-1 and PRV-3 suggest species-specific differences in salmonid RBC responses to PRV infection, offering new insights into the pathogenesis of PRV-3 in coho salmon and PRV-1 in Atlantic salmon.

### 4.1. Description of the PRV Inoculums

The PRV-1b variant used in this study was previously sequenced in 2017 and classified within the PRV-1b variant group, which is considered among the most virulent [35]. The PRV-3a variant was partially sequenced and isolated from coho salmon exhibiting clinical signs of jaundice syndrome [15]. At the molecular level, these viruses display 81.8% nucleotide similarity in the S1 segment and 76.7% similarity in the M2 segment. Notably, PRV infections display host-specific effects, with PRV-1 causing mild hemoglobin reductions in Atlantic salmon [5], while PRV-3 triggers severe anemia in coho salmon [14].

### 4.2. Replication Kinetics of PRV in RBC

Wessel et al. [24] initially showed that PRV-1 infects Atlantic salmon RBC ex vivo. In their initial experiments, viral load increased from an initial Ct value of 22.3 to a peak of 14.6 ± 0.9 at 14 days post-infection (dpi). Additionally, they observed an increase in viral load in the supernatant fraction and in viral protein production over 21 days of infection. In the present study, an increase in viral load was observed from 1 to 14 dpi in csRBC infected with PRV-3 and asRBC infected with PRV-1 (Figure 1). Earlier experiments by Wessel et al. [24] indicated a higher replication rate of PRV-1 in asRBC. This discrepancy may stem from differences in the processing of the PRV-1 inoculum; in the reference study, the virus was not purified via ultracentrifugation, potentially resulting in a higher number of infective virions per cell. Although PRV-1 and PRV-3 are phylogenetically distinct and infect different salmonid host species, the present results reaffirm that PRV targets salmonid RBC. This information is key to perfecting an ex vivo culture platform that allows PRV propagation. Currently, studies with PRV rely on in vivo propagation, requiring numerous salmonid individuals and takes 4–6 weeks [7]. No cell lines are reported to support PRV replication [22]. It is imperative to conduct screenings of cell lines using not only Norwegian PRV-1 isolates but also PRV-3 isolates, utilizing cell lines derived from homotypic hosts.

### 4.3. Cell Viability and Apoptosis

Haematological disorders caused by PRV are likely to be initiated by interactions between RBC and the virus. Understanding the host–pathogen interaction at the cellular level provides essential insights into the mechanisms underlying PRV diseases. No RBC lysis due to PRV-1 infection has been found in previous experiments [24], but this had not previously been tested in csRBC infected with PRV-3 (Figure 2). The Trypan blue method may not detect if viral infection induces an early phase of apoptosis or slightly decreased viability. In this study, we confirmed that PRV infection does not cause hemolysis in salmonid RBC. This was demonstrated using Trypan blue exclusion and propidium iodide staining in two independent trials. However, ex vivo culture over time led to increased cellular senescence in control cells. Specifically, after 14 days of culture, control csRBCs showed subtle morphological changes such as partial rounding and mild cytoplasmic condensation, consistent with early signs of senescence. These changes occurred in the absence of any treatment or infection and were observed at a lower frequency compared to PRV-3-infected cells. Furthermore, Trypan blue staining revealed that cell viability in the control group was not 100%, suggesting that extended culture conditions alone can induce some degree of cellular stress and senescence. Cellular senescence in RBC is characterized by several morphological and biochemical changes, including a shift from an elliptical to a more rounded shape, decreased size, loss of cytoplasm and cellular content, and increased membrane rigidity [36]. A previous study reported morphological changes associated with cellular senescence in RBC of rainbow trout exposed to mechanical stress, drastic temperature variations, and viral infections [37]. The lifespan of RBC culminates in macrophage-mediated clearance of senescent cells through erythrophagocytosis, a process involving the exposure of phosphatidylserine (PS) on the erythrocyte membrane, which is a well-established mechanism [36].

Fish RBC, being nucleated and metabolically active, offer an opportunity to quantify metabolic activity using the Alamar blue fluorometric method [38]. To our knowledge, this is the first study to apply the Alamar blue method to salmonid RBC, and no toxicity data are available for this assay in these cells. Our results indicate that Alamar blue was non-toxic to RBC, unlike the MTT assay.

Early apoptosis in RBC was measured using commercial Annexin V kits. This method has been used in the fish RBC infected with *Aeromonas hydrophila* [39] and in rainbow trout RBC exposed to different temperatures ex vivo [37]. In this study, only PRV-3-infected coho salmon RBC showed a higher percentage of cells with decreased metabolic activity (viability) and early apoptotic cells, related to the higher PRV-3 viral load at 14 dpi.

The advantage for PRV virions when host cells become metabolically non-viable and undergo apoptosis remains unclear. However, the observed cellular senescence associated with PRV infection may be attributed to the nonstructural p13 protein, encoded by the bicistronic S1 segment. This integral membrane protein localizes to the Golgi apparatus and exhibits cytotoxicity in vitro [40]. Two hypotheses have been proposed regarding PRV virion release, where the p13 protein plays a key role: (a) as a viroporin, facilitating passive virion release through membrane pores, or (b) by inducing apoptosis, leading to immune-mediated destruction of infected cells and subsequent virion release [40]. Although the viroporin hypothesis remains plausible, this study supports the apoptosis-mediated release hypothesis for PRV-3 in csRBC, suggesting that virion dissemination occurs through immune recognition of PS residues on RBC membranes. This mechanism has been demonstrated for other salmonid viruses, including infectious salmon anemia virus (ISAV) [41] and infectious pancreatic necrosis virus (IPNV) [42], but not for PRV-1.

The apoptotic pathway in salmonid RBC has been poorly studied. In fish, the key apoptotic pathway consists of a cascade of cysteine proteases known as caspases. Caspases participate in apoptosis as both initiators and executioners. In fish, *casp8* and *casp9* are commonly found as initiators, while *casp3* and *casp6* serve as executioners [39,43]. Additionally, the apoptosis pathway involves members of the *bcl2* family, which regulate mitochondrial permeability and function. These members can be categorized into two distinct groups: anti-apoptotic members such as Bcl-2, which prevent cell death, and pro-apoptotic elements like *bax*. The ratio of mRNA expression of *bax* to *bcl2* is used to determine the life or death of cells in response to an apoptotic stimulus [44]. In this study, *casp8* and the ratio *bax*/*bcl2* are upregulated in csRBC, implying the potential involvement of these genes in the induction of apoptosis by PRV-3. The unexpected downregulation of apoptotic *casp3* and *casp9* in csRBC reveals a distinctive mechanism through which PRV-3 may instigate apoptosis, potentially to evade the host immune system or promote its own replication and spread within the host organism. Interestingly, in Atlantic salmon RBC infected with purified PRV-1, the PI3K-Akt pathway exhibited significantly higher transcript levels at 1 dpi compared to non-susceptible cell lines, as demonstrated by Tsoulia et al. [45].

In this context, we hypothesize that the hematocrit reduction and severe anemia observed in coho salmon with jaundice syndrome and with HSMI-like disease caused by PRV-3 [14,21] are due to RBC destruction via apoptosis rather than lysis. Considering that spleen, liver, and heart are the primary affected organs in these diseases, erythrophagocytosis in the spleen may facilitate viral release and subsequent infection of secondary organs. To validate this hypothesis, in vivo infection studies with PRV-3a in naïve coho salmon and systemic analyses are necessary.

### 4.4. Antiviral Immune Response in RBC

The interaction between csRBC and PRV-3 showed immune antiviral responses related to the IFN-I pathway, similar to what was previously reported for asRBC infected with PRV-1 ex vivo [24]. The IFN-I pathway is the primary innate immune mechanism through which vertebrates combat viral infections. The dsRNA genome of PRV is recognized by specific receptors (PPRs). This triggers responses that induce IFN-I production and the expression of numerous antiviral effector molecules [46]. In this study, dsRNA recognition was analyzed through gene expression of the retinoic acid-inducible gene I (*rig-i*), a member of the *rig-i*-like receptor (RLR) family. The *rig-i* detects viral RNA via its RNA helicase and C-terminal domains, initiating interactions with mitochondrial antiviral signaling proteins to activate downstream immune responses. This activation leads to IFN production and the upregulation of antiviral genes such as *mx*, *pkr*, *isg15*, and *viperin*, which inhibit viral replication and spread [46]. Using Poly I:C, a synthetic dsRNA, as a positive control, we confirmed increased *rig-i* expression in csRBC. Notably, significant upregulation was observed from 3 dpi in PRV-infected groups compared to the positive control, suggesting heightened dsRNA recognition during PRV infection.

Antiviral effectors such as *mx* and *pkr* were upregulated in both PRV-infected groups and Poly I:C-treated cells at 3, 7, and 14 dpi, following the earlier upregulation of *ifnα* at 1 dpi in PRV-infected cells. Among these, the highest expression levels were observed in experiment I for PRV-3, indicating a stronger immune response in csRBC compared to asRBC against PRV-1. These findings are consistent with previous research on asRBC infected with PRV-1b, which showed significant upregulation of *ifnα*, *mx*, *rig-i*, and *pkr* transcripts at 1 and 7 dpi [24]. Transcriptomic studies on PRV-1-infected RBC have also demonstrated significant upregulation of *ifnα* and *irf1* six weeks post-exposure in cohabitant RBC, with *mx* and *rig-i* induction from week seven. Similar findings have been reported in Atlantic salmon, where *ifnα*, *viperin*, *isg15*, *pkr*, and *ifnγ* transcripts peaked between 4 and 6 weeks post-challenge [29]. In rainbow trout infected with PRV-3, *mx* and *viperin* expression increased in the spleen during peak viral loads in the blood and heart of cohabitants six weeks post-challenge.

However, when purified PRV-1 was used in this study, the immune response was not significantly different from that induced by Poly I:C. Tsoulia et al. [45] showed that the exposure of Atlantic salmon RBC to purified PRV-1 for 24 h induced a characteristic antiviral response of intermediate strength, albeit delayed compared to the non-susceptible salmon head kidney (SHK-1) cell line to PRV-1. This response was characterized by elevated expression of the interferon regulatory factor gene *IRF1*, but without upregulation of *mx*, *isg15*, and *viperin* [45]. In this study, when purified PRV-1 was employed, the cellular response did not significantly differ from basal levels and was comparable to that of Poly I:C stimulation. The use of purified viruses in viral infection assays offers a crucial advantage by providing a controlled experimental environment to study the precise effects of the pathogen on the host cell. Purified virus preparations ensure that observed outcomes are directly attributable to the virus itself, minimizing the influence of potential confounding factors present in homogenized samples, such as cellular debris and contaminants.

Interestingly, our results showed that coho salmon RBC could function as antigen-presenting cells with an upregulation of major histocompatibility complex class I (*mhc-i*) (Figure 7A). *mhc-i* molecules are found on the surface of nucleated cells and present peptides derived from endogenous proteins, including viral proteins, on CD8+ T-cells. When a T-helper cell recognizes a viral peptide presented by an *mhc-i* molecule, it is activated and can then kill the infected cells by inducing apoptosis or secretion of antiviral cytokines [47]. Upregulation of *mhc-i* transcripts in salmonid RBC has been reported when rainbow trout RBC were exposed to viral hemorrhagic septicemia virus (VHSv) [28]. Transcriptomic analysis of PRV-1 infected Atlantic salmon RBC showed that the second largest gene group to be induced was genes involved in viral antigen presentation, including MHC class I antigens. Similarly, RBC from rock bream infected with rock bream iridovirus (RBIV) demonstrated upregulation of *mhc-i*-related pathways [48].

Collectively, these findings and prior studies highlight the relationship between the robust pro-inflammatory antiviral immune response in salmonids and the development of PRV-associated diseases. The induction of this response depends on the PRV genotype, host species, and environmental factors. For instance, low-virulence PRV-1a isolates elicit weaker immune responses and are not associated with disease in Columbia River Pacific salmon, whereas high-virulence PRV-1b isolates induce strong antiviral responses, resulting in HSMI in Atlantic salmon. In contrast, PRV-3a has not been isolated from healthy coho salmon or rainbow trout, while PRV-3b has been found in clinically healthy brown trout but is also associated with disease in rainbow trout and coho salmon [49,50,51]. Additionally, reduced water temperatures have been shown to enhance PRV-3b replication and heart pathology in rainbow trout [52]. Currently, it has not been described whether the virulence of PRV-3 variants is associated with polymorphisms in the S1, S4, M2, L1, and L2 segments as in PRV-1, or simply due to the ability of host RBC to support high PRV-3 viral loads.

The distinct outcomes of PRV-1 in Atlantic salmon and PRV-3 in coho salmon underscore the complex host–virus dynamics. In Atlantic salmon, PRV-1 infection is persistent without inducing apoptosis or anemia, potentially due to the virus’ ability to evade immune clearance. Conversely, PRV-3 in coho salmon triggers a robust immune response, leading to apoptosis and potential anemia. The precise mechanisms underlying PRV evasion of host immune responses remain unclear but may involve the σ3 protein, which can bind dsRNA and potentially inhibit IFN signaling, as observed in mammalian orthoreovirus (MRV) [53]. Understanding these dynamics is essential for developing effective aquaculture disease management strategies and advancing virological research in fish populations.

## Figures and Tables

**Table 1:** Primers used for RT-qPCR and qPCR analyses. Fw: forward sequence (5′—3′); Rv: reverse sequence (5′—3′).

Gene	Sequence	Amplicon Size	Reference
Immune genes			
*ifnα* (both experiments)	Fw: ACTGAAACGCTACTTCAAGAAGTTGA	104	[24]
	Rv: GCAGATGACGTTTTGTCTCTTTCCT		
*rig-i* (both experiments)	Fw: ACGCCTTGAAGAGCTGGATA	89	[24]
	Rv: CTGGCTGGACTTGTGTCCTC		
*pkr* (both experiments)	Fw: CAGGATGCAACACCATCATC	162	[24]
	Rv: GGTCTTGACCGGTGACATCT		
*mx* (experiment I)	Fw: GATGCTGCACCTCAAGTCCTATTA	82	[24]
	Rv: CACCAGGTAGCGGATCACCAT		
*mx* (experiment II)	Fw: GGTGATAGGGGACCAGAGT	173	[32]
	Rv: CTCCTCACGGTCTTGGTAGC		
*isg15* (experiment I)	Fw: GGCCTGCATTCAGGATCTAA	120	[29]
	Rv: TACAGTCTCACCAGGCACCA		
*isg15* (experiment II)	Fw: ATATCTACTGAACATATATCTATCATGGAACTC	150	[32]
	Rv: CCTCTGCTTTGTTGTGGCCACTT		
*viperin* (both experiments)	Fw: AGCAATGGCAGCATGATCAG	101	[6]
	Rv: TGGTTGGTGTCCTCGTCAAAG		
*mhc-i*	Fw: CCAGAGGATGTATGGTTGTGAG	124	[28]
	Rv: TGGAGCGATCCATGTCTTTGTC		
*ef1α* (experiment II)	Fw: TGCCCCTCCAGGATGTCTAC	57	[18]
	Rv: CACGGCCCACAGGTACTG		
*β-actinA* (experiment I)	Fw: GGACTTTGAGCAGGAGATGG	91	This study
	Rv: ATGATGGAGTTGTAGGTGGTC		
Piscine orthoreovirus detection			
PRV3-S1 (experiment I)	Fw: AATGACAGACCAGACCGACG	115	This study
	Rv: CCATCCAGCCACTGAAACCA		
PRV1-S1 (experiment II)	Fw: TGCGTCCTGCGTATGGCACC	143	[24]
	Rv: GGCTGGCATGCCCGAATAGCA		
	Probe: 6FAM- ATCACAACGCCTACCT –MGBNFQ		
Apoptosis genes in csRBC			
*casp8*	Fw: GTCAGGGGATCCAGTGTGTG	100	This study
	Rv: TCTCTGCACAGGAAGCACAG		
*casp9*	Fw: CCAGGGCAACAGGAAGAGTT	135	This study
	Rv: TGGACCCTGTGCGGTTATTC		
*casp3*	Fw: GGATGAGTTGGGCCAGTGAA	99	This study
	Rv: TTGATGTGTGCAGAGGGTCC		
*bax*	Fw: TGGCCTTCTACATAGTGTTCTTTC	59	This study
	Rv: GTGGCTTCGTTTTGCTCGTT		
*bcl2*	Fw: TTCTCAGCTACCAACCCTGG	84	This study
	Rv: ACGTCAGTTCCTTACCGACAC		

## Data Availability

The original contributions presented in this study are included in the article/Supplementary Materials. Further inquiries can be directed to the corresponding author.

## Supplementary Materials

The following supporting information can be downloaded at: https://www.mdpi.com/article/10.3390/microorganisms13051167/s1, Figure S1. Standardization of the Alamar Blue method in coho salmon RBC (csRBC) ex vivo. The principle of the method is based on the reduction of resazurin (non-fluorescent blue) to resorufin (fluorescent pink) by metabolically viable cells. A. csRBC (1x105 cells/well) were plated in a 96-well plate and exposed to various concentrations of DMSO. Cells were loaded with AlamarBlue Reagent (10 μL), incubated at 15 °C for 72 h. The plates were measured at 540 nm Excitation/590 nm Emission (Fluorescence). B. Cell viability curve and IC50 value for the cytotoxicity effect of DMSO in coho salmon RBC (n = 3); Figure S2. Validation of housekeeping genes. A. β-actinA gene as reference for qPCR gene expression analysis during PRV infection of coho salmon RBC ex vivo. B. ef-1αgene as reference for qPCR gene expression analysis during PRV infection of Atlantic salmon RBC ex vivo. The mean Ct values of the housekeeping genes are presented at different time points after infection with PRV and immunostimulation with Poly I:C (50 μg/mL); Figure S3. Flow cytometry compensation controls for the apoptosis assay in csRBC (A, B and C) and asRBC (D, E and F). A. Unstained control for csRBC (FITC-A vs. PE-A). B. Propidium iodide, PE stained (dead control) for csRBC. C. Annexin V, FITC stained (apoptosis control) for csRBC. D. Unstained control for asRBC (FITC-A vs. PE-A); E. Propidium iodide, PE stained (dead control) for asRBC. F) Annexin V, FITC stained (apoptosis control) for asRBC. Graphs are representative of one biological replicate for each experiment; Figure S4. Visualization of RBC labeled with Annexin V (A, B) and Propidium Iodide (C) in immunofluorescence microscopy for confirmation of the Annexin V-Alexa Fluor 484 Kit in csRBC (A, C) and asRBC (B). Magnification of 20X in A, C. Magnification of 40X in B; Figure S5. Non-morphological changes in Atlantic salmon RBC (asRBC) ex vivo at 14 dpi. Images were taken using a phase contrast microscope at 20X amplification. A. Control cells. B. Poly I:C (50 µg/mL) treatment. C. PRV-1 treatment; Figure S6. Flow cytometry apoptosis assay using Annexin V-PI dyes in RBC infected with PRV at 14 dpi in experiment I (A, B, C) and experiment II (D, E, F). A. Control group of csRBC. B. csRBC + Poly I:C (50 µg/mL). C. csRBC + PRV-3. D. Control group of asRBC. E. asRBC + Poly I:C (50 µg/mL). F. asRBC + PRV-1. In all graphs, Q1 corresponds to cells stained with PI (necrotic cells), Q2 corresponds to cells stained with PI and Annexin V (late apoptotic cells), Q3 corresponds to cells stained with Annexin V (early apoptotic cells), and Q4 corresponds to cells without staining (viable cells). The graphs represent one biological replicate for each experiment; Table S1. Summary of statistics *p*-values for fold change of transcripts analyzed in coho salmon red blood cells (csRBC) and Atlantic salmon red blood cells (asRBC) ex vivo. Significant differences (*p* < 0.05) are represented in bold, two-way ANOVA.
