# Supplementary material for: Comparative Analysis of PRV-1 in Atlantic Salmon and PRV-3 in Coho Salmon: Host-Specific Immune Responses and Apoptosis in Red Blood Cells
Source: Microorganisms. 2025 May 21;13(5):1167. doi: 10.3390/microorganisms13051167 (PMC12113769; doi:10.3390/microorganisms13051167)
Supplement: Supplementary file 1 [file microorganisms-13-01167-s001.zip › microorganisms-3582051-supplementary.pdf]

Comparative analysis of PRV-1 in Atlantic salmon and PRV-3 in coho salmon: Host-Specific Immune Responses and Apoptosis in Red Blood Cells.

Supplementary material

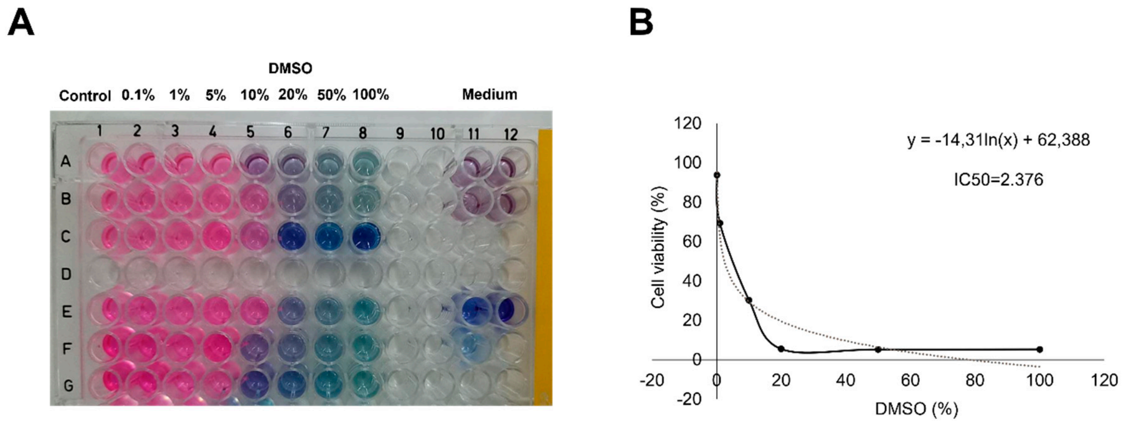

Figure S1. Standardization of the Alamar Blue method in coho salmon RBC (csRBC) *ex vivo*. The principle of the method is based on the reduction of resazurin (non-fluorescent blue) to resorufin (fluorescent pink) by metabolically viable cells. **A.** csRBC (1x10^5 cells/well) were plated in a 96-well plate and exposed to various concentrations of DMSO. Cells were loaded with AlamarBlue Reagent (10 µl), incubated at 15°C for 72 hours. The plates were measured at 540 nm Excitation / 590 nm Emission (Fluorescence). **B.** Cell viability curve and IC50 value for the cytotoxicity effect of DMSO in coho salmon RBC (n = 3).

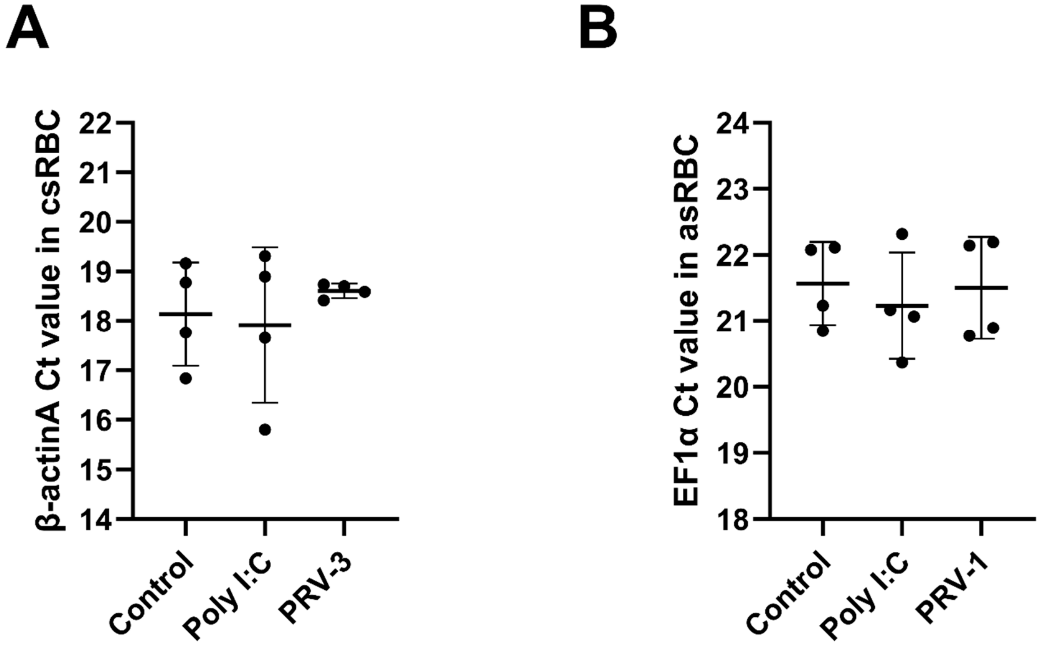

Figure S2. Validation of housekeeping genes. **A.** *β-actinA* gene as reference for qPCR gene expression analysis during PRV infection of coho salmon RBC *ex vivo*. **B.** *ef-1α*gene as reference for qPCR gene expression analysis during PRV infection of Atlantic salmon RBC *ex vivo.* The mean Ct values of the housekeeping genes are presented at different time points after infection with PRV and immunostimulation with Poly I:C (50 µg/ml).

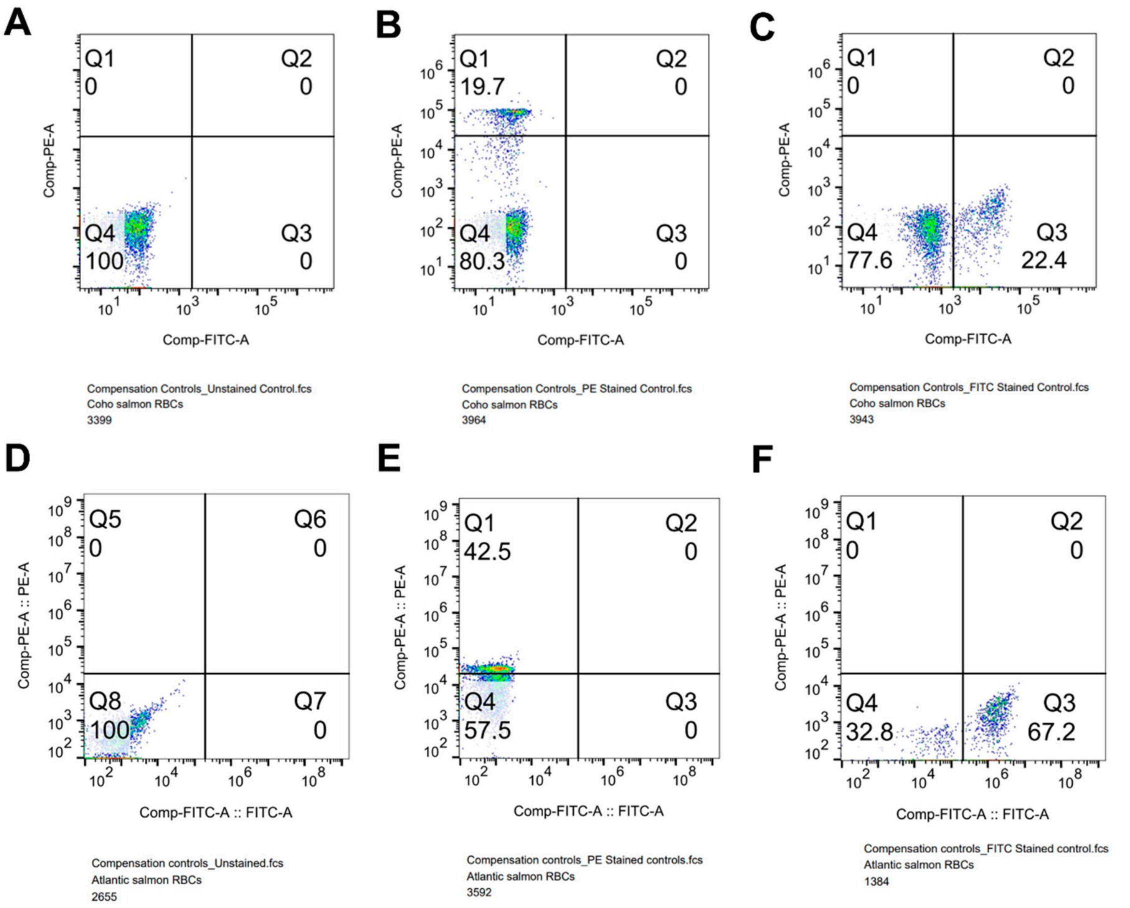

Figure S3. Flow cytometry compensation controls for the apoptosis assay in csRBC (**A, B and C)** and asRBC (**D, E and F**). **A.** Unstained control for csRBC (FITC-A vs PE-A). **B.** Propidium iodide, PE stained (dead control) for csRBC. **C.** Annexin V, FITC stained (apoptosis control) for csRBC. **D.** Unstained control for asRBC (FITC-A vs PE-A); **E.** Propidium iodide, PE stained (dead control) for asRBC. **F)** Annexin V, FITC stained (apoptosis control) for asRBC. Graphs are representative of one biological replicate for each experiment.

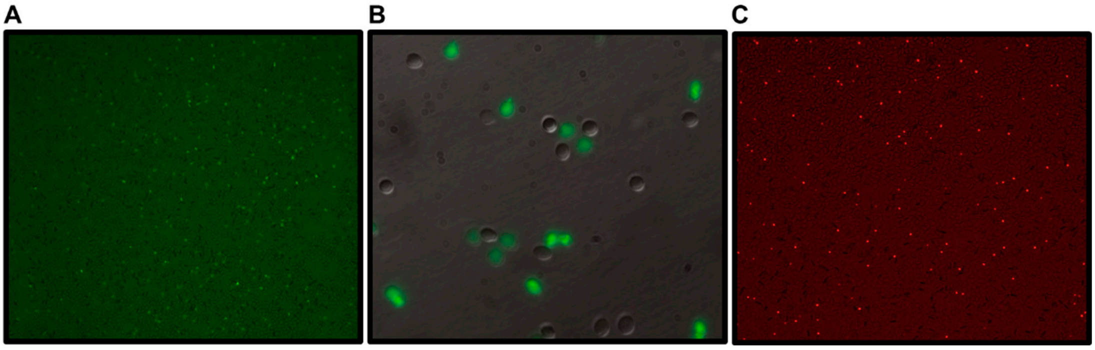

Figure S4. Visualization of RBC labeled with Annexin V (**A, B**) and Propidium Iodide (**C**) in immunofluorescence microscopy for confirmation of the Annexin V-Alexa Fluor 484 Kit in csRBC (**A, C**) and asRBC (**B**). Magnification of 20X in **A, C**. Magnification of 40X in **B**.

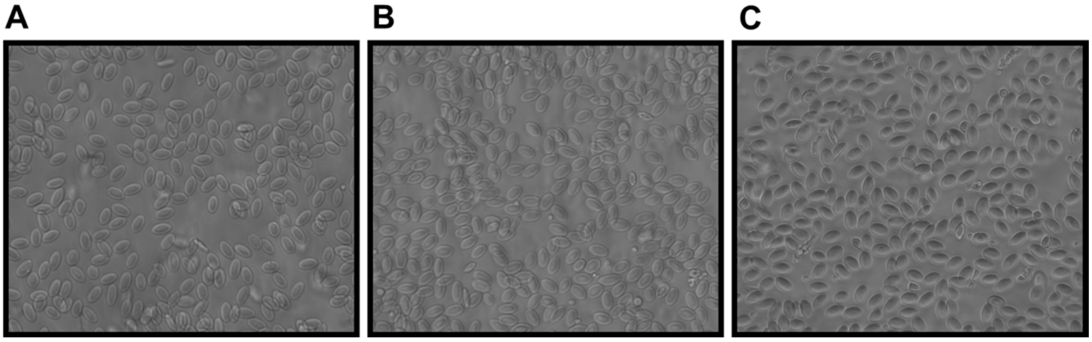

Figure S5. Non-morphological changes in Atlantic salmon RBC (asRBC) *ex vivo* at 14 dpi. Images were taken using a phase contrast microscope at 20X amplification. **A.** Control cells. **B.** Poly I:C (50 µg/ml) treatment. **C.** PRV-1 treatment.

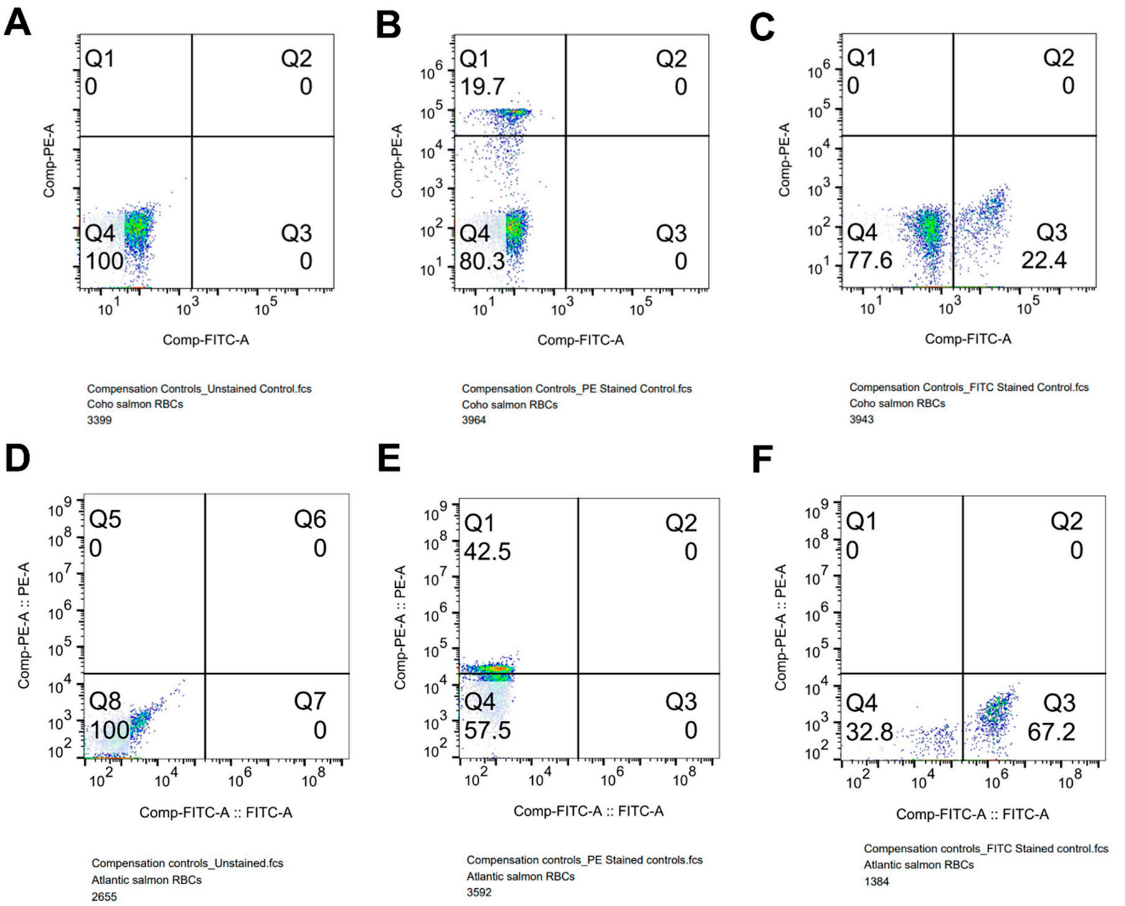

Figure S6. Flow cytometry apoptosis assay using Annexin V-PI dyes in RBC infected with PRV at 14 dpi in experiment I (**A, B, C**) and experiment II (**D, E, F**). **A.** Control group of csRBC. **B.** csRBC + Poly I:C (50 μg/ml). **C.** csRBC + PRV-3. **D.** Control group of asRBC. **E.** asRBC + Poly I:C (50 μg/ml). **F.** asRBC + PRV-1. In all graphs, Q1 corresponds to cells stained with PI (necrotic cells), Q2 corresponds to cells stained with PI and Annexin V (late apoptotic cells), Q3 corresponds to cells stained with Annexin V (early apoptotic cells), and Q4 corresponds to cells without staining (viable cells). The graphs represent one biological replicate for each experiment.

Table S1. Summary of statistics *p*-values for fold change of transcripts analyzed in coho salmon red blood cells (csRBC) and Atlantic salmon red blood cells (asRBC) *ex vivo*. Significant differences (*p<0.05*) are represented in bold, two-way ANOVA.

**Coho salmon red blood cells *ex vivo* (csRBC)**					
**Treatment group comparison**	**Transcript**	**Days post-infection (dpi)**			
		**1**	**3**	**7**	**14**
Control vs Poly I:C	*ifnα*	0.4581	0.5895	0.1725	0.4054
Control vs PRV-3	*ifnα*	0.6097	0.1977	0.487	0.1718
Poly I:C vs PRV-3	*ifnα*	0.6566	0.2593	0.783	0.3385
Control vs Poly I:C	*rig-i*	0.6118	0.6135	0.9035	0.0718
Control vs PRV-3	*rig-i*	0.3559	**0.0367**	0.1672	0.1625
Poly I:C vs PRV-3	*rig-i*	0.9035	**0.0238**	0.1492	0.3119
Control vs Poly I:C	*mx*	0.5796	0.6751	0.1789	0.5156
Control vs PRV-3	*mx*	0.3555	**0.0199**	**0.0266**	0.2653
Poly I:C vs PRV-3	*mx*	0.8325	**0.0169**	**0.0205**	0.2819
Control vs Poly I:C	*pkr*	0.7363	0.4003	0.3635	0.1106
Control vs PRV-3	*pkr*	0.2307	0.0949	0.15	**0.0384**
Poly I:C vs PRV-3	*pkr*	0.4299	0.1637	0.1622	**0.0448**

Control vs Poly I:C	*isg15*	0.4448	0.212	0.3081	0.2103
Control vs PRV-3	*isg15*	0.4034	0.241	0.291	0.0716
Poly I:C vs PRV-3	*isg15*	0.5407	0.3991	0.5314	0.0829
Control vs Poly I:C	*viperin*	0.7249	0.5146	0.1831	0.6006
Control vs PRV-3	*viperin*	0.2917	0.0665	0.3635	0.9989
Poly I:C vs PRV-3	*viperin*	0.6059	0.075	0.4242	0.8972
Control vs Poly I:C	*mhc-I*	0.8114	0.7976	0.9313	0.942
Control vs PRV-3	*mhc-I*	0.2007	**0.0486**	0.1992	0.4479
Poly I:C vs PRV-3	*mhc-I*	0.6041	**0.0341**	0.2051	0.5396
Control vs Poly I:C	*casp8*	0.4899	0.5471	0.2159	0.7936
Control vs PRV-3	*casp8*	0.3014	**0.0196**	0.4653	0.3087
Poly I:C vs PRV-3	*casp8*	0.7591	**0.0066**	0.6858	0.8003
Control vs Poly I:C	*casp9*	0.6711	0.9999	**0.0285**	**0.0412**
Control vs PRV-3	*casp9*	0.3107	0.1831	0.4638	**0.0281**
Poly I:C vs PRV-3	*casp9*	0.992	0.5025	0.7501	0.1366
Control vs Poly I:C	*casp3*	0.3987	0.7037	0.9968	0.6219
Control vs PRV-3	*casp3*	0.4421	0.3936	**0.0029**	**0.0013**
Poly I:C vs PRV-3	*casp3*	0.9476	0.4381	0.3144	0.1751
Control vs Poly I:C	*bax/bcl2*	0.1863	0.7832	0.2409	0.2788
Control vs PRV-3	*bax/bcl2*	0.3407	0.906	**0.0001**	0.1879
Poly I:C vs PRV-3	*bax/bcl2*	0.9508	0.9377	0.1167	0.2053
**Atlantic salmon red blood cells *ex vivo* (asRBC)**					

Treatment group comparison	Transcript	Days post-infection (dpi)			
		1	3	7	14
Control vs Poly I:C	*ifnα*	0.1279	0.2136	0.3844	0.2547
Control vs PRV-1	*ifnα*	0.8682	0.6536	0.9311	0.1181
Poly I:C vs PRV-1	*ifnα*	0.1017	0.9956	0.3935	0.6269
Control vs Poly I:C	*rig-i*	0.1654	0.2842	0.2501	0.255
Control vs PRV-1	*rig-i*	0.7656	0.3295	0.5673	0.2304
Poly I:C vs PRV-1	*rig-i*	0.1737	0.8038	0.296	0.326
Control vs Poly I:C	*mx*	0.1006	0.1491	0.1987	0.2928
Control vs PRV-1	*mx*	0.7324	0.1671	0.4379	0.2477
Poly I:C vs PRV-1	*mx*	0.1019	0.22	0.2307	0.3171
Control vs Poly I:C	*pkr*	0.3763	0.2726	0.2403	0.2736
Control vs PRV-1	*pkr*	0.9775	0.2255	0.4951	0.1607
Poly I:C vs PRV-1	pkr	0.3782	0.5081	0.2832	0.3076
Control vs Poly I:C	*isg15*	0.37	0.0662	0.1353	0.3236
Control vs PRV-1	*isg15*	0.6777	0.4954	0.3626	0.2639
Poly I:C vs PRV-1	*isg15*	0.3813	0.1017	0.144	0.3351
Control vs Poly I:C	*viperin*	0.333	0.1669	0.2149	0.2846
Control vs PRV-1	*viperin*	0.4972	0.4269	0.4396	0.1536
Poly I:C vs PRV-1	*viperin*	0.346	0.4044	0.2349	0.3194
Control vs Poly I:C	*mhc-I*	0.9246	0.8629	0.6675	0.4974
Control vs PRV-1	*mhc-I*	0.9016	0.6235	0.9845	0.4843

Poly I:C vs PRV-1	*mhc-I*	0.9999	0.9499	0.8703	0.9511
